# Ethyl 1,5-diphenyl-1*H*-pyrazole-4-carboxyl­ate

**DOI:** 10.1107/S1600536810031223

**Published:** 2010-08-11

**Authors:** Hoong-Kun Fun, Ching Kheng Quah, B. Chandrakantha, Arun M. Isloor, Prakash Shetty

**Affiliations:** a X-ray Crystallography Unit, School of Physics, Universiti Sains Malaysia, 11800 USM, Penang, Malaysia; b Department of Chemistry, Manipal Institute of Technology, Manipal 576 104, India; c Organic Chemistry Division, Department of Chemistry, National Institute of Technology–Karnataka, Surathkal, Mangalore 575 025, India; d Department of Printing, Manipal Institute of Technology, Manipal 576 104, India

## Abstract

The asymmetric unit of the title compound, C_18_H_16_N_2_O_2_, contains two independent mol­ecules (*A* and *B*). In mol­ecule *A*, the pyrazole ring is inclined at angles of 48.86 (6) and 60.80 (6)° with respect to the two phenyl rings; the corresponding angles for mol­ecule *B* are 46.86 (6) and 58.63 (6)°. In the crystal, mol­ecules of type *A* are linked into sheets parallel to (001) *via* weak C—H⋯O hydrogen bonds, whereas the mol­ecules of type *B* are linked into chains along [010] *via* weak C—H⋯O hydrogen bonds.

## Related literature

For general background to and the biological activity of pyrazole derivatives, see: Isloor *et al.* (2009 ▶); Lambert & Fowler (2005 ▶); Lan *et al.* (1999 ▶). For related structures, see: Fun *et al.* (2009 ▶; 2010 ▶). For the stability of the temperature controller used in the data collection, see: Cosier & Glazer (1986 ▶). For standard bond-length data, see: Allen *et al.* (1987 ▶). For hydrogen-bond motifs, see: Bernstein *et al.* (1995 ▶).
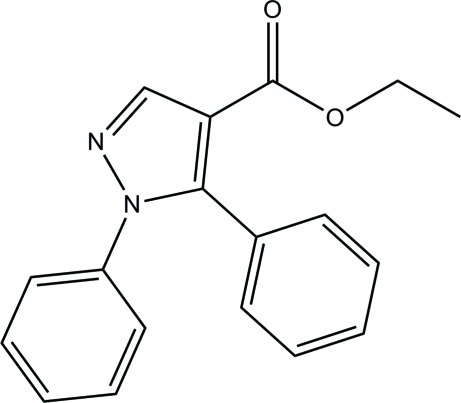


## Experimental

### 

#### Crystal data

C_18_H_16_N_2_O_2_
*M* *_r_* = 292.33
Triclinic, 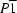

*a* = 9.2015 (8) Å
*b* = 10.4638 (9) Å
*c* = 16.9332 (15) Å
α = 97.515 (2)°
β = 104.605 (2)°
γ = 104.578 (2)°
*V* = 1493.7 (2) Å^3^
*Z* = 4
Mo *K*α radiation
μ = 0.09 mm^−1^
*T* = 100 K
0.24 × 0.23 × 0.21 mm

#### Data collection

Bruker SMART APEXII DUO CCD area-detector diffractometer
Absorption correction: multi-scan (*SADABS*; Bruker, 2009 ▶) *T*_min_ = 0.980, *T*_max_ = 0.983
35429 measured reflections
10300 independent reflections
7846 reflections with *I* > 2σ(*I*)
*R*_int_ = 0.044

#### Refinement

*R*[*F* ^2^ > 2σ(*F* ^2^)] = 0.046
*wR*(*F* ^2^) = 0.128
*S* = 1.03
10300 reflections
399 parameters
H-atom parameters constrained
Δρ_max_ = 0.43 e Å^−3^
Δρ_min_ = −0.28 e Å^−3^

### 

Data collection: *APEX2* (Bruker, 2009 ▶); cell refinement: *SAINT* (Bruker, 2009 ▶); data reduction: *SAINT*; program(s) used to solve structure: *SHELXTL* (Sheldrick, 2008 ▶); program(s) used to refine structure: *SHELXTL*; molecular graphics: *SHELXTL*; software used to prepare material for publication: *SHELXTL* and *PLATON* (Spek, 2009 ▶).

## Supplementary Material

Crystal structure: contains datablocks global, I. DOI: 10.1107/S1600536810031223/lh5108sup1.cif

Structure factors: contains datablocks I. DOI: 10.1107/S1600536810031223/lh5108Isup2.hkl

Additional supplementary materials:  crystallographic information; 3D view; checkCIF report

## Figures and Tables

**Table 1:** Hydrogen-bond geometry (Å, °)

*D*—H⋯*A*	*D*—H	H⋯*A*	*D*⋯*A*	*D*—H⋯*A*
C3*A*—H3*AA*⋯O2*A*^i^	0.93	2.43	3.3591 (16)	177
C3*B*—H3*BA*⋯O2*B*^i^	0.93	2.46	3.3829 (16)	172
C13*A*—H13*A*⋯O2*A*^ii^	0.93	2.59	3.2622 (17)	129

Symmetry codes: (i) 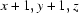; (ii) 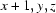.

**Fig. 1.**
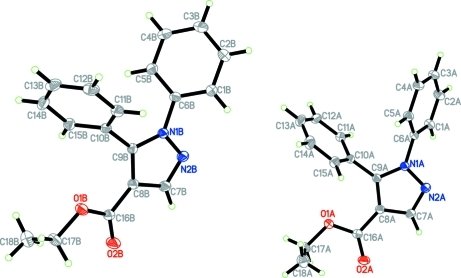
The molecular structure of the title compound showing 50% probability displacement ellipsoids for non-H atoms and the atom-numbering scheme.

**Fig. 2.**
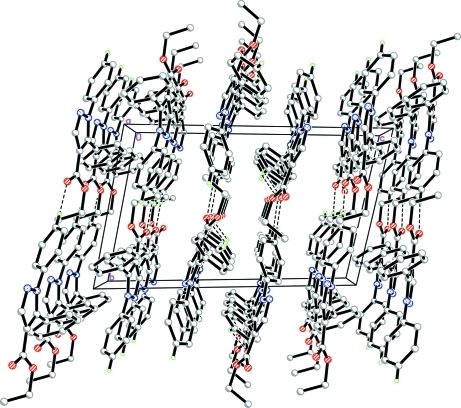
Part of the crystal structure of the title compound, viewed approximately along the a axis. H atoms not involved in hydrogen bonds (dashed lines) have been omitted for clarity.

**Fig. 3.**
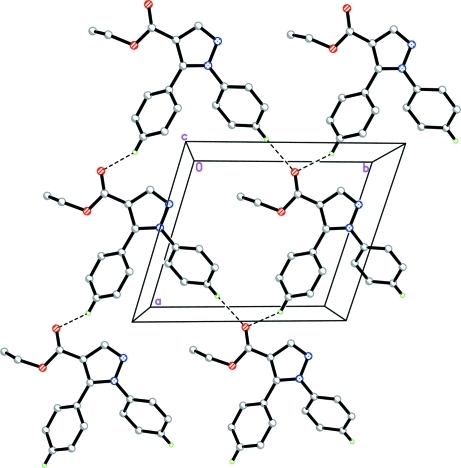
The crystal packing of molecules of type A, viewed along the c axis. H atoms not involved in hydrogen bonds (dashed lines) have been omitted for clarity.

## supplementary crystallographic information

### Comment

Pyrazole and its derivatives represent one of the most active classes of compounds possessing a wide spectrum of biological activities. During the past years, considerable evidences have been accumulated to demonstrate the efficacy of pyrazole derivatives including antibacterial (Isloor *et al.*, 2009), antifungal (Lambert & Fowler, 2005), herbicidal (Lan *et al.*, 1999), insecticidal and other biological activities. In view of the potential importance of pyrazole derivatives, we have synthesized the title compound and its crystal structure is presented herein.

The title compound (Fig. 1) contains two indpendent molecules (*A* and *B*) in the asymmetric unit, with similar geometries. Each molecule consists of two phenyl rings and an ethyl carboxylate moiety attached to the pyrazole ring. In molecule *A*, the pyrazole ring (N1A/N2A/C7A-C9A) is inclined at angles of 48.86 (6) and 60.80 (6)° with respect to the C1A-C6A and C10A-C15A phenyl rings, respectively. The correspondening angles for molecule *B* are 46.86 (6) and 58.63 (6)°. Bond lengths and angles are within normal ranges, and comparable to closely related structures (Fun *et al.*, 2009; 2010).

In the crystal packing (Fig. 2), molecules of type *A* are linked into sheets (Fig. 3) parallel to (001) *via* C3A–H3AA···O2A^i^ and C13A–H13A···O2A^ii^ hydrogen bonds whereas the molecules *B* are linked into one-dimensional chains along [010] *via* a C3B–H3BA···O2B^i^ hydrogen bonds.

### Experimental

A mixture of ethyl-3-(dimethylamino)-2-(phenylcarbonyl)prop-2-enoate (2.0 g, 0.0080 mol) and phenyl hydrazine (0.95 g, 0.0088 mol) in absolute ethanol (20 ml) was refluxed for 2 h. Reaction completion was monitored through thin layer chromatography and the reaction mixture was evaporated under reduced pressure. The residue was stirred with 1.5N HCl and the solid separated was filtered and dried under vacuum. The solid obtained was purified by column chromatography using silica gel 60-120 mesh size and petroleum ether: ethyl acetate as eluent to afford title compound as colourless crystals (2.0g, 86.9 %); melting point: 400-405 K.

### Refinement

All H atoms were positioned geometrically and refined using a riding model with C-H = 0.93-0.97 Å and *U*_iso_(H) = 1.2 or 1.5 *U*_eq_(C). A rotating-group model was applied for the methyl groups.

### Crystal data

**Table:**

C_18_H_16_N_2_O_2_	*Z* = 4
*M**_r_* = 292.33	*F*(000) = 616
Triclinic, *P*1	*D*_x_ = 1.300 Mg m^−^^3^
Hall symbol: -P 1	Mo *K*α radiation, λ = 0.71073 Å
*a* = 9.2015 (8) Å	Cell parameters from 9846 reflections
*b* = 10.4638 (9) Å	θ = 2.5–31.9°
*c* = 16.9332 (15) Å	µ = 0.09 mm^−^^1^
α = 97.515 (2)°	*T* = 100 K
β = 104.605 (2)°	Block, colourless
γ = 104.578 (2)°	0.24 × 0.23 × 0.21 mm
*V* = 1493.7 (2) Å^3^	

### Data collection

**Table:**

Bruker SMART APEXII DUO CCD area-detector diffractometer	10300 independent reflections
Radiation source: fine-focus sealed tube	7846 reflections with *I* > 2σ(*I*)
graphite	*R*_int_ = 0.044
φ and ω scans	θ_max_ = 32.1°, θ_min_ = 2.1°
Absorption correction: multi-scan (*SADABS*; Bruker, 2009)	*h* = −13→13
*T*_min_ = 0.980, *T*_max_ = 0.983	*k* = −15→15
35429 measured reflections	*l* = −25→25

### Refinement

**Table:**

Refinement on *F*^2^	Primary atom site location: structure-invariant direct methods
Least-squares matrix: full	Secondary atom site location: difference Fourier map
*R*[*F*^2^ > 2σ(*F*^2^)] = 0.046	Hydrogen site location: inferred from neighbouring sites
*wR*(*F*^2^) = 0.128	H-atom parameters constrained
*S* = 1.03	*w* = 1/[σ^2^(*F*_o_^2^) + (0.0568*P*)^2^ + 0.3537*P*] where *P* = (*F*_o_^2^ + 2*F*_c_^2^)/3
10300 reflections	(Δ/σ)_max_ = 0.001
399 parameters	Δρ_max_ = 0.43 e Å^−^^3^
0 restraints	Δρ_min_ = −0.28 e Å^−^^3^

### Special details

**Table:**

Experimental. The crystal was placed in the cold stream of an Oxford Cyrosystems Cobra open-flow nitrogen cryostat (Cosier & Glazer, 1986) operating at 100.0 (1) K.
Geometry. All esds (except the esd in the dihedral angle between two l.s. planes) are estimated using the full covariance matrix. The cell esds are taken into account individually in the estimation of esds in distances, angles and torsion angles; correlations between esds in cell parameters are only used when they are defined by crystal symmetry. An approximate (isotropic) treatment of cell esds is used for estimating esds involving l.s. planes.
Refinement. Refinement of F^2^ against ALL reflections. The weighted R-factor wR and goodness of fit S are based on F^2^, conventional R-factors R are based on F, with F set to zero for negative F^2^. The threshold expression of F^2^ > 2sigma(F^2^) is used only for calculating R-factors(gt) etc. and is not relevant to the choice of reflections for refinement. R-factors based on F^2^ are statistically about twice as large as those based on F, and R- factors based on ALL data will be even larger.

### Fractional atomic coordinates and isotropic or equivalent isotropic displacement parameters (Å^2^)

**Table:**

	*x*	*y*	*z*	*U*_iso_*/*U*_eq_	
O1A	0.35565 (9)	0.55673 (8)	0.40840 (5)	0.02192 (16)	
O2A	0.10538 (10)	0.56385 (8)	0.36818 (6)	0.02587 (18)	
N1A	0.45702 (10)	0.94866 (9)	0.36135 (5)	0.01681 (16)	
N2A	0.30699 (11)	0.95931 (9)	0.34270 (6)	0.01975 (17)	
C1A	0.59279 (14)	1.18876 (11)	0.40504 (7)	0.0206 (2)	
H1AA	0.5214	1.1983	0.4340	0.025*	
C2A	0.71181 (15)	1.30011 (11)	0.40425 (7)	0.0244 (2)	
H2AA	0.7195	1.3851	0.4325	0.029*	
C3A	0.81991 (14)	1.28608 (12)	0.36156 (7)	0.0241 (2)	
H3AA	0.9000	1.3611	0.3619	0.029*	
C4A	0.80757 (13)	1.15950 (12)	0.31842 (7)	0.0236 (2)	
H4AA	0.8794	1.1499	0.2897	0.028*	
C5A	0.68803 (13)	1.04687 (11)	0.31798 (7)	0.0205 (2)	
H5AA	0.6790	0.9621	0.2888	0.025*	
C6A	0.58225 (12)	1.06269 (10)	0.36174 (6)	0.01680 (18)	
C7A	0.21906 (13)	0.83992 (11)	0.34605 (7)	0.01922 (19)	
H7AA	0.1106	0.8170	0.3360	0.023*	
C8A	0.30951 (12)	0.75140 (10)	0.36682 (6)	0.01709 (18)	
C9A	0.46399 (12)	0.82515 (10)	0.37630 (6)	0.01586 (18)	
C10A	0.61394 (12)	0.79251 (10)	0.39942 (6)	0.01714 (18)	
C11A	0.73369 (13)	0.86837 (11)	0.47083 (7)	0.0213 (2)	
H11A	0.7182	0.9383	0.5044	0.026*	
C12A	0.87630 (14)	0.83944 (13)	0.49183 (8)	0.0270 (2)	
H12A	0.9554	0.8892	0.5400	0.032*	
C13A	0.90112 (15)	0.73682 (14)	0.44132 (8)	0.0299 (3)	
H13A	0.9971	0.7185	0.4553	0.036*	
C14A	0.78255 (16)	0.66135 (13)	0.36982 (8)	0.0287 (3)	
H14A	0.7996	0.5929	0.3357	0.034*	
C15A	0.63871 (14)	0.68781 (11)	0.34916 (7)	0.0223 (2)	
H15A	0.5588	0.6359	0.3019	0.027*	
C16A	0.24476 (12)	0.61555 (10)	0.38013 (6)	0.01793 (19)	
C17A	0.29923 (14)	0.41764 (11)	0.41795 (7)	0.0229 (2)	
H17A	0.2071	0.4069	0.4374	0.027*	
H17B	0.3801	0.3979	0.4594	0.027*	
C18A	0.25821 (16)	0.32054 (13)	0.33655 (8)	0.0295 (3)	
H18A	0.2355	0.2298	0.3456	0.044*	
H18B	0.3453	0.3387	0.3139	0.044*	
H18C	0.1676	0.3309	0.2981	0.044*	
O1B	−0.03838 (10)	0.61507 (8)	0.09374 (5)	0.02177 (16)	
O2B	−0.23012 (11)	0.65343 (9)	0.14598 (6)	0.0317 (2)	
N1B	0.11226 (10)	1.04064 (9)	0.13619 (5)	0.01698 (16)	
N2B	−0.01563 (11)	1.06169 (9)	0.15725 (6)	0.02083 (18)	
C1B	0.18999 (13)	1.25412 (11)	0.09434 (7)	0.0210 (2)	
H1BA	0.0849	1.2424	0.0668	0.025*	
C2B	0.30546 (15)	1.37023 (12)	0.09588 (8)	0.0253 (2)	
H2BA	0.2772	1.4370	0.0698	0.030*	
C3B	0.46273 (14)	1.38736 (12)	0.13605 (7)	0.0248 (2)	
H3BA	0.5396	1.4651	0.1369	0.030*	
C4B	0.50423 (13)	1.28742 (12)	0.17498 (7)	0.0234 (2)	
H4BA	0.6095	1.2980	0.2013	0.028*	
C5B	0.38976 (13)	1.17171 (11)	0.17499 (7)	0.0202 (2)	
H5BA	0.4178	1.1056	0.2019	0.024*	
C6B	0.23336 (12)	1.15597 (10)	0.13444 (6)	0.01708 (18)	
C7B	−0.10204 (13)	0.93979 (11)	0.15709 (7)	0.0208 (2)	
H7BA	−0.1970	0.9225	0.1696	0.025*	
C8B	−0.03368 (12)	0.83895 (10)	0.13579 (6)	0.01782 (19)	
C9B	0.10638 (12)	0.90767 (10)	0.12275 (6)	0.01598 (18)	
C10B	0.22948 (12)	0.86100 (10)	0.09737 (6)	0.01613 (18)	
C11B	0.26595 (13)	0.89043 (11)	0.02510 (7)	0.0201 (2)	
H11B	0.2110	0.9379	−0.0075	0.024*	
C12B	0.38418 (14)	0.84894 (12)	0.00174 (7)	0.0251 (2)	
H12B	0.4064	0.8671	−0.0470	0.030*	
C13B	0.46887 (14)	0.78059 (12)	0.05093 (8)	0.0268 (2)	
H13B	0.5492	0.7544	0.0358	0.032*	
C14B	0.43344 (14)	0.75128 (12)	0.12286 (8)	0.0245 (2)	
H14B	0.4903	0.7055	0.1559	0.029*	
C15B	0.31331 (13)	0.79004 (11)	0.14585 (7)	0.0199 (2)	
H15B	0.2889	0.7687	0.1935	0.024*	
C16B	−0.11071 (13)	0.69496 (11)	0.12682 (7)	0.0197 (2)	
C17B	−0.10723 (15)	0.47049 (11)	0.08556 (7)	0.0235 (2)	
H17C	−0.0773	0.4212	0.0428	0.028*	
H17E	−0.2210	0.4486	0.0683	0.028*	
C18B	−0.05319 (17)	0.42793 (13)	0.16657 (8)	0.0311 (3)	
H18G	−0.0918	0.3315	0.1583	0.047*	
H18D	−0.0928	0.4690	0.2073	0.047*	
H18E	0.0597	0.4560	0.1859	0.047*	

### Atomic displacement parameters (Å^2^)

**Table:**

	*U*^11^	*U*^22^	*U*^33^	*U*^12^	*U*^13^	*U*^23^
O1A	0.0188 (4)	0.0176 (3)	0.0286 (4)	0.0037 (3)	0.0057 (3)	0.0084 (3)
O2A	0.0173 (4)	0.0204 (4)	0.0391 (5)	0.0021 (3)	0.0108 (3)	0.0055 (3)
N1A	0.0150 (4)	0.0155 (4)	0.0214 (4)	0.0049 (3)	0.0070 (3)	0.0050 (3)
N2A	0.0159 (4)	0.0206 (4)	0.0254 (4)	0.0074 (3)	0.0081 (3)	0.0060 (3)
C1A	0.0236 (5)	0.0179 (5)	0.0218 (5)	0.0057 (4)	0.0093 (4)	0.0046 (4)
C2A	0.0277 (6)	0.0175 (5)	0.0249 (5)	0.0028 (4)	0.0074 (4)	0.0025 (4)
C3A	0.0209 (5)	0.0214 (5)	0.0268 (5)	0.0005 (4)	0.0055 (4)	0.0085 (4)
C4A	0.0188 (5)	0.0248 (5)	0.0293 (5)	0.0050 (4)	0.0107 (4)	0.0092 (4)
C5A	0.0197 (5)	0.0193 (5)	0.0242 (5)	0.0056 (4)	0.0092 (4)	0.0055 (4)
C6A	0.0166 (5)	0.0155 (4)	0.0182 (4)	0.0042 (4)	0.0047 (4)	0.0054 (3)
C7A	0.0155 (5)	0.0208 (5)	0.0225 (5)	0.0056 (4)	0.0072 (4)	0.0049 (4)
C8A	0.0159 (4)	0.0163 (4)	0.0190 (4)	0.0040 (4)	0.0059 (4)	0.0036 (3)
C9A	0.0160 (4)	0.0149 (4)	0.0170 (4)	0.0045 (3)	0.0056 (3)	0.0033 (3)
C10A	0.0162 (4)	0.0170 (4)	0.0206 (4)	0.0056 (4)	0.0072 (4)	0.0072 (3)
C11A	0.0188 (5)	0.0211 (5)	0.0235 (5)	0.0046 (4)	0.0059 (4)	0.0067 (4)
C12A	0.0166 (5)	0.0333 (6)	0.0309 (6)	0.0055 (5)	0.0047 (4)	0.0148 (5)
C13A	0.0202 (5)	0.0411 (7)	0.0404 (7)	0.0161 (5)	0.0152 (5)	0.0234 (6)
C14A	0.0319 (6)	0.0323 (6)	0.0357 (6)	0.0201 (5)	0.0199 (5)	0.0140 (5)
C15A	0.0230 (5)	0.0224 (5)	0.0244 (5)	0.0093 (4)	0.0095 (4)	0.0051 (4)
C16A	0.0174 (5)	0.0169 (4)	0.0189 (4)	0.0038 (4)	0.0063 (4)	0.0026 (3)
C17A	0.0258 (6)	0.0164 (5)	0.0259 (5)	0.0041 (4)	0.0070 (4)	0.0078 (4)
C18A	0.0352 (7)	0.0243 (6)	0.0296 (6)	0.0108 (5)	0.0096 (5)	0.0034 (4)
O1B	0.0222 (4)	0.0157 (3)	0.0279 (4)	0.0037 (3)	0.0112 (3)	0.0026 (3)
O2B	0.0239 (4)	0.0226 (4)	0.0545 (6)	0.0052 (3)	0.0226 (4)	0.0100 (4)
N1B	0.0144 (4)	0.0150 (4)	0.0222 (4)	0.0043 (3)	0.0071 (3)	0.0031 (3)
N2B	0.0163 (4)	0.0199 (4)	0.0289 (5)	0.0067 (3)	0.0101 (4)	0.0041 (3)
C1B	0.0177 (5)	0.0192 (5)	0.0256 (5)	0.0055 (4)	0.0050 (4)	0.0049 (4)
C2B	0.0264 (6)	0.0192 (5)	0.0312 (6)	0.0053 (4)	0.0096 (5)	0.0088 (4)
C3B	0.0229 (5)	0.0205 (5)	0.0293 (5)	0.0005 (4)	0.0120 (4)	0.0023 (4)
C4B	0.0157 (5)	0.0252 (5)	0.0259 (5)	0.0027 (4)	0.0063 (4)	0.0003 (4)
C5B	0.0172 (5)	0.0203 (5)	0.0220 (5)	0.0053 (4)	0.0045 (4)	0.0033 (4)
C6B	0.0157 (4)	0.0148 (4)	0.0197 (4)	0.0026 (3)	0.0064 (4)	0.0014 (3)
C7B	0.0176 (5)	0.0202 (5)	0.0259 (5)	0.0056 (4)	0.0094 (4)	0.0033 (4)
C8B	0.0160 (5)	0.0162 (4)	0.0217 (5)	0.0040 (4)	0.0071 (4)	0.0037 (3)
C9B	0.0156 (4)	0.0152 (4)	0.0171 (4)	0.0050 (3)	0.0045 (3)	0.0030 (3)
C10B	0.0140 (4)	0.0140 (4)	0.0194 (4)	0.0029 (3)	0.0058 (3)	0.0012 (3)
C11B	0.0202 (5)	0.0190 (5)	0.0210 (5)	0.0043 (4)	0.0078 (4)	0.0035 (4)
C12B	0.0239 (5)	0.0238 (5)	0.0267 (5)	0.0017 (4)	0.0142 (4)	−0.0002 (4)
C13B	0.0179 (5)	0.0253 (5)	0.0347 (6)	0.0045 (4)	0.0110 (4)	−0.0052 (4)
C14B	0.0196 (5)	0.0240 (5)	0.0290 (6)	0.0102 (4)	0.0042 (4)	0.0005 (4)
C15B	0.0195 (5)	0.0196 (5)	0.0213 (5)	0.0071 (4)	0.0062 (4)	0.0034 (4)
C16B	0.0168 (5)	0.0181 (5)	0.0247 (5)	0.0045 (4)	0.0075 (4)	0.0046 (4)
C17B	0.0270 (6)	0.0147 (4)	0.0274 (5)	0.0030 (4)	0.0095 (4)	0.0028 (4)
C18B	0.0393 (7)	0.0271 (6)	0.0311 (6)	0.0134 (5)	0.0127 (5)	0.0093 (5)

### Geometric parameters (Å, °)

**Table:**

O1A—C16A	1.3402 (13)	O1B—C16B	1.3420 (13)
O1A—C17A	1.4620 (13)	O1B—C17B	1.4587 (13)
O2A—C16A	1.2123 (13)	O2B—C16B	1.2156 (13)
N1A—C9A	1.3630 (13)	N1B—C9B	1.3651 (13)
N1A—N2A	1.3728 (12)	N1B—N2B	1.3719 (12)
N1A—C6A	1.4330 (13)	N1B—C6B	1.4311 (13)
N2A—C7A	1.3235 (14)	N2B—C7B	1.3216 (14)
C1A—C2A	1.3879 (15)	C1B—C6B	1.3882 (15)
C1A—C6A	1.3906 (14)	C1B—C2B	1.3905 (16)
C1A—H1AA	0.9300	C1B—H1BA	0.9300
C2A—C3A	1.3935 (17)	C2B—C3B	1.3893 (18)
C2A—H2AA	0.9300	C2B—H2BA	0.9300
C3A—C4A	1.3893 (17)	C3B—C4B	1.3887 (18)
C3A—H3AA	0.9300	C3B—H3BA	0.9300
C4A—C5A	1.3927 (15)	C4B—C5B	1.3905 (15)
C4A—H4AA	0.9300	C4B—H4BA	0.9300
C5A—C6A	1.3917 (14)	C5B—C6B	1.3865 (15)
C5A—H5AA	0.9300	C5B—H5BA	0.9300
C7A—C8A	1.4142 (15)	C7B—C8B	1.4100 (14)
C7A—H7AA	0.9300	C7B—H7BA	0.9300
C8A—C9A	1.3936 (14)	C8B—C9B	1.3947 (14)
C8A—C16A	1.4668 (14)	C8B—C16B	1.4643 (14)
C9A—C10A	1.4743 (15)	C9B—C10B	1.4749 (14)
C10A—C11A	1.3940 (15)	C10B—C15B	1.3959 (15)
C10A—C15A	1.3986 (14)	C10B—C11B	1.3996 (14)
C11A—C12A	1.3903 (16)	C11B—C12B	1.3922 (15)
C11A—H11A	0.9300	C11B—H11B	0.9300
C12A—C13A	1.3851 (19)	C12B—C13B	1.3862 (19)
C12A—H12A	0.9300	C12B—H12B	0.9300
C13A—C14A	1.389 (2)	C13B—C14B	1.3898 (18)
C13A—H13A	0.9300	C13B—H13B	0.9300
C14A—C15A	1.3882 (17)	C14B—C15B	1.3926 (15)
C14A—H14A	0.9300	C14B—H14B	0.9300
C15A—H15A	0.9300	C15B—H15B	0.9300
C17A—C18A	1.5010 (16)	C17B—C18B	1.5015 (17)
C17A—H17A	0.9700	C17B—H17C	0.9700
C17A—H17B	0.9700	C17B—H17E	0.9700
C18A—H18A	0.9600	C18B—H18G	0.9600
C18A—H18B	0.9600	C18B—H18D	0.9600
C18A—H18C	0.9600	C18B—H18E	0.9600
			
C16A—O1A—C17A	116.10 (9)	C16B—O1B—C17B	115.69 (8)
C9A—N1A—N2A	112.87 (8)	C9B—N1B—N2B	112.76 (8)
C9A—N1A—C6A	128.77 (9)	C9B—N1B—C6B	129.09 (9)
N2A—N1A—C6A	118.36 (8)	N2B—N1B—C6B	118.10 (8)
C7A—N2A—N1A	104.39 (9)	C7B—N2B—N1B	104.38 (8)
C2A—C1A—C6A	118.80 (10)	C6B—C1B—C2B	119.22 (10)
C2A—C1A—H1AA	120.6	C6B—C1B—H1BA	120.4
C6A—C1A—H1AA	120.6	C2B—C1B—H1BA	120.4
C1A—C2A—C3A	120.78 (10)	C3B—C2B—C1B	120.56 (11)
C1A—C2A—H2AA	119.6	C3B—C2B—H2BA	119.7
C3A—C2A—H2AA	119.6	C1B—C2B—H2BA	119.7
C4A—C3A—C2A	119.73 (10)	C4B—C3B—C2B	119.46 (11)
C4A—C3A—H3AA	120.1	C4B—C3B—H3BA	120.3
C2A—C3A—H3AA	120.1	C2B—C3B—H3BA	120.3
C3A—C4A—C5A	120.24 (10)	C3B—C4B—C5B	120.59 (11)
C3A—C4A—H4AA	119.9	C3B—C4B—H4BA	119.7
C5A—C4A—H4AA	119.9	C5B—C4B—H4BA	119.7
C6A—C5A—C4A	119.18 (10)	C6B—C5B—C4B	119.24 (10)
C6A—C5A—H5AA	120.4	C6B—C5B—H5BA	120.4
C4A—C5A—H5AA	120.4	C4B—C5B—H5BA	120.4
C1A—C6A—C5A	121.27 (10)	C5B—C6B—C1B	120.92 (10)
C1A—C6A—N1A	118.48 (9)	C5B—C6B—N1B	120.56 (9)
C5A—C6A—N1A	120.24 (9)	C1B—C6B—N1B	118.47 (9)
N2A—C7A—C8A	111.99 (9)	N2B—C7B—C8B	112.23 (9)
N2A—C7A—H7AA	124.0	N2B—C7B—H7BA	123.9
C8A—C7A—H7AA	124.0	C8B—C7B—H7BA	123.9
C9A—C8A—C7A	105.26 (9)	C9B—C8B—C7B	105.21 (9)
C9A—C8A—C16A	130.70 (10)	C9B—C8B—C16B	131.97 (9)
C7A—C8A—C16A	123.90 (9)	C7B—C8B—C16B	122.74 (9)
N1A—C9A—C8A	105.49 (9)	N1B—C9B—C8B	105.43 (9)
N1A—C9A—C10A	122.05 (9)	N1B—C9B—C10B	122.19 (9)
C8A—C9A—C10A	132.43 (9)	C8B—C9B—C10B	132.36 (9)
C11A—C10A—C15A	119.52 (10)	C15B—C10B—C11B	119.26 (9)
C11A—C10A—C9A	119.93 (9)	C15B—C10B—C9B	120.66 (9)
C15A—C10A—C9A	120.52 (10)	C11B—C10B—C9B	120.06 (9)
C12A—C11A—C10A	120.01 (11)	C12B—C11B—C10B	120.32 (11)
C12A—C11A—H11A	120.0	C12B—C11B—H11B	119.8
C10A—C11A—H11A	120.0	C10B—C11B—H11B	119.8
C13A—C12A—C11A	120.29 (12)	C13B—C12B—C11B	120.14 (11)
C13A—C12A—H12A	119.9	C13B—C12B—H12B	119.9
C11A—C12A—H12A	119.9	C11B—C12B—H12B	119.9
C12A—C13A—C14A	119.96 (11)	C12B—C13B—C14B	119.81 (10)
C12A—C13A—H13A	120.0	C12B—C13B—H13B	120.1
C14A—C13A—H13A	120.0	C14B—C13B—H13B	120.1
C15A—C14A—C13A	120.17 (11)	C13B—C14B—C15B	120.44 (11)
C15A—C14A—H14A	119.9	C13B—C14B—H14B	119.8
C13A—C14A—H14A	119.9	C15B—C14B—H14B	119.8
C14A—C15A—C10A	120.02 (11)	C14B—C15B—C10B	120.00 (10)
C14A—C15A—H15A	120.0	C14B—C15B—H15B	120.0
C10A—C15A—H15A	120.0	C10B—C15B—H15B	120.0
O2A—C16A—O1A	123.97 (10)	O2B—C16B—O1B	124.08 (10)
O2A—C16A—C8A	123.00 (10)	O2B—C16B—C8B	122.69 (10)
O1A—C16A—C8A	113.02 (9)	O1B—C16B—C8B	113.22 (9)
O1A—C17A—C18A	110.68 (9)	O1B—C17B—C18B	111.06 (10)
O1A—C17A—H17A	109.5	O1B—C17B—H17C	109.4
C18A—C17A—H17A	109.5	C18B—C17B—H17C	109.4
O1A—C17A—H17B	109.5	O1B—C17B—H17E	109.4
C18A—C17A—H17B	109.5	C18B—C17B—H17E	109.4
H17A—C17A—H17B	108.1	H17C—C17B—H17E	108.0
C17A—C18A—H18A	109.5	C17B—C18B—H18G	109.5
C17A—C18A—H18B	109.5	C17B—C18B—H18D	109.5
H18A—C18A—H18B	109.5	H18G—C18B—H18D	109.5
C17A—C18A—H18C	109.5	C17B—C18B—H18E	109.5
H18A—C18A—H18C	109.5	H18G—C18B—H18E	109.5
H18B—C18A—H18C	109.5	H18D—C18B—H18E	109.5
			
C9A—N1A—N2A—C7A	−0.07 (11)	C9B—N1B—N2B—C7B	0.18 (12)
C6A—N1A—N2A—C7A	179.13 (9)	C6B—N1B—N2B—C7B	−177.38 (9)
C6A—C1A—C2A—C3A	−0.58 (17)	C6B—C1B—C2B—C3B	0.86 (17)
C1A—C2A—C3A—C4A	0.76 (18)	C1B—C2B—C3B—C4B	−0.09 (17)
C2A—C3A—C4A—C5A	−0.20 (18)	C2B—C3B—C4B—C5B	−0.87 (17)
C3A—C4A—C5A—C6A	−0.50 (17)	C3B—C4B—C5B—C6B	1.04 (16)
C2A—C1A—C6A—C5A	−0.14 (16)	C4B—C5B—C6B—C1B	−0.24 (15)
C2A—C1A—C6A—N1A	−178.67 (10)	C4B—C5B—C6B—N1B	−177.47 (9)
C4A—C5A—C6A—C1A	0.68 (16)	C2B—C1B—C6B—C5B	−0.70 (16)
C4A—C5A—C6A—N1A	179.18 (10)	C2B—C1B—C6B—N1B	176.58 (10)
C9A—N1A—C6A—C1A	−132.33 (11)	C9B—N1B—C6B—C5B	−46.46 (15)
N2A—N1A—C6A—C1A	48.61 (13)	N2B—N1B—C6B—C5B	130.65 (10)
C9A—N1A—C6A—C5A	49.12 (15)	C9B—N1B—C6B—C1B	136.25 (11)
N2A—N1A—C6A—C5A	−129.93 (11)	N2B—N1B—C6B—C1B	−46.64 (13)
N1A—N2A—C7A—C8A	0.13 (11)	N1B—N2B—C7B—C8B	−0.39 (12)
N2A—C7A—C8A—C9A	−0.14 (12)	N2B—C7B—C8B—C9B	0.45 (13)
N2A—C7A—C8A—C16A	175.97 (9)	N2B—C7B—C8B—C16B	−176.57 (10)
N2A—N1A—C9A—C8A	−0.01 (11)	N2B—N1B—C9B—C8B	0.09 (12)
C6A—N1A—C9A—C8A	−179.11 (9)	C6B—N1B—C9B—C8B	177.32 (10)
N2A—N1A—C9A—C10A	−178.57 (9)	N2B—N1B—C9B—C10B	178.45 (9)
C6A—N1A—C9A—C10A	2.33 (15)	C6B—N1B—C9B—C10B	−4.31 (16)
C7A—C8A—C9A—N1A	0.09 (11)	C7B—C8B—C9B—N1B	−0.30 (11)
C16A—C8A—C9A—N1A	−175.66 (10)	C16B—C8B—C9B—N1B	176.32 (11)
C7A—C8A—C9A—C10A	178.43 (10)	C7B—C8B—C9B—C10B	−178.43 (11)
C16A—C8A—C9A—C10A	2.68 (19)	C16B—C8B—C9B—C10B	−1.8 (2)
N1A—C9A—C10A—C11A	59.02 (14)	N1B—C9B—C10B—C15B	121.72 (11)
C8A—C9A—C10A—C11A	−119.10 (13)	C8B—C9B—C10B—C15B	−60.41 (16)
N1A—C9A—C10A—C15A	−119.47 (11)	N1B—C9B—C10B—C11B	−56.76 (14)
C8A—C9A—C10A—C15A	62.42 (15)	C8B—C9B—C10B—C11B	121.11 (13)
C15A—C10A—C11A—C12A	−0.23 (16)	C15B—C10B—C11B—C12B	0.22 (16)
C9A—C10A—C11A—C12A	−178.73 (10)	C9B—C10B—C11B—C12B	178.72 (10)
C10A—C11A—C12A—C13A	1.08 (17)	C10B—C11B—C12B—C13B	−1.36 (17)
C11A—C12A—C13A—C14A	−0.71 (18)	C11B—C12B—C13B—C14B	1.23 (17)
C12A—C13A—C14A—C15A	−0.50 (18)	C12B—C13B—C14B—C15B	0.04 (17)
C13A—C14A—C15A—C10A	1.35 (18)	C13B—C14B—C15B—C10B	−1.18 (17)
C11A—C10A—C15A—C14A	−0.98 (16)	C11B—C10B—C15B—C14B	1.04 (16)
C9A—C10A—C15A—C14A	177.51 (10)	C9B—C10B—C15B—C14B	−177.45 (10)
C17A—O1A—C16A—O2A	5.26 (15)	C17B—O1B—C16B—O2B	−3.28 (16)
C17A—O1A—C16A—C8A	−176.13 (8)	C17B—O1B—C16B—C8B	178.27 (9)
C9A—C8A—C16A—O2A	−179.16 (11)	C9B—C8B—C16B—O2B	175.52 (12)
C7A—C8A—C16A—O2A	5.79 (16)	C7B—C8B—C16B—O2B	−8.35 (18)
C9A—C8A—C16A—O1A	2.22 (15)	C9B—C8B—C16B—O1B	−6.00 (17)
C7A—C8A—C16A—O1A	−172.83 (9)	C7B—C8B—C16B—O1B	170.12 (10)
C16A—O1A—C17A—C18A	83.99 (12)	C16B—O1B—C17B—C18B	−81.78 (13)

### Hydrogen-bond geometry (Å, °)

**Table:**

*D*—H···*A*	*D*—H	H···*A*	*D*···*A*	*D*—H···*A*
C3A—H3AA···O2A^i^	0.93	2.43	3.3591 (16)	177
C3B—H3BA···O2B^i^	0.93	2.46	3.3829 (16)	172
C13A—H13A···O2A^ii^	0.93	2.59	3.2622 (17)	129

Symmetry codes: (i) *x*+1, *y*+1, *z*; (ii) *x*+1, *y*, *z*.
